# Effects of Electrospinning Parameters on the Microstructure of PVP/TiO_2_ Nanofibers

**DOI:** 10.3390/nano11061616

**Published:** 2021-06-20

**Authors:** Wan-Tae Kim, Dong-Cheol Park, Wan-Hee Yang, Churl-Hee Cho, Won-Youl Choi

**Affiliations:** 1 Department of Advanced Materials Engineering, Gangneung-Wonju National University, Gangneung, Gangwon 25457, Korea; kwt3809@gmail.com (W.-T.K.); cufe2000@naver.com (D.-C.P.); 2 Research Institute for Dental Engineering, Gangneung-Wonju National University, Gangneung, Gangwon 25457, Korea; 3 WITH M-TECH Co., Ltd., Suwon, Gyeonggi 16229, Korea; dangchan74@empas.com; 4 Graduate School of Energy Science and Technology, Chungnam National University, Daejeon 34134, Korea

**Keywords:** electrospinning, TiO_2_, PVP, nanofiber

## Abstract

Titanium dioxide has excellent chemical, electrical, and optical properties, as well as good chemical stability. For that reason, it is widely used in many fields of study and industry, such as photocatalysts, organic solar cells, sensors, dental implants, and other applications. Many nanostructures of TiO_2_ have been reported, and electrospinning is an efficient practical technique that has a low cost and high efficiency. In various studies on improving performance, the researchers created nanofibers with suitable microstructures by changing various properties and the many process parameters that can be controlled. In this study, PVP/TiO_2_ nanofibers were fabricated by the electrospinning process. The diameters of the nanofibers were controlled by various parameters. To understand the effects on the diameter of the nanofibers, various process parameters were controlled: the molecular weight and concentration of the polymers, deionized water, applied voltage, fluid velocity, and concentration of titanium precursor. The average diameter of the PVP nanofibers was controlled in a range of 42.3 nm to 633.0 nm. The average diameter of the PVP/TiO_2_ nanofibers was also controlled in a range of 63.5 nm to 186.0 nm after heat treatment.

## 1. Introduction

Titanium dioxide has excellent chemical, electrical, and optical properties, as well as chemical stability. For that reason, it is widely used in many fields of study and industry, such as photocatalysts, organic solar cells, sensors, dental implants, and other applications [1,2,3,4,5,6,7,8,9,10,11,12,13,14]. It is a very useful material and is commonly used for photocatalysts due to its photoactivity, high stability, low cost, and safety for the environment and humans [7,15,16]. Many nanostructures of TiO_2_ have been reported with improved properties, for example, nanoparticles, nanofibers, and nanotubes [4,5,7,9,10,11,12,17,18,19,20,21,22,23]. These nanostructures are fabricated by the sol-gel method, hydrothermal treatment, anodic oxidation, and electrospinning [13,17,18,23,24,25,26,27,28]. With their large specific area and semiconductor properties, electrospun TiO_2_ nanofibers are being researched in many fields, such as photocatalysts, solar cells, and metal oxide gas sensors [4,9,18,29,30,31,32,33,34,35]. Electrospinning is an efficient practical technique that is low cost and has a high efficiency, and many studies have reported the production of various nanofibers [4,9,18,19,20,21,22,23,36,37,38]. In electrospinning, the precursor solution flows at a constant rate through a pump, in such a way as to create a continuous nanofiber, and then electrodes are connected to the inflowing electrospinning solution, while other electrodes are connected to the appliance plate. At this time, if a high voltage is applied, it is emitted in a conical shape by surface tension at the end of the electrospinning solution [23,26,27,28,39]. The charge is subsequently stored in the electrospinning solution, and mutual repulsion causes the cone to be radiatively stretched to a jet when the surface tension of the electrospinning solution is exceeded. In the radiation-stretched electrospinning solution, volatilization of the solvent occurs before it collects in the plate, which can result in disorderly arranged nanofibers in the plate. The diameter of nanofibers is controlled by the influence of these various instabilities. When applying nanofibers to be used in various studies, to improve performance, nanofibers with suitable microstructure are needed to change various properties, and many process parameters need to be controlled.

In this study, polyvinyl pyrrolidone (PVP)/TiO_2_ nanofibers were fabricated by an electrospinning process. To understand their effects on the diameter of the nanofibers, various process parameters were controlled: the molecular weight and concentration of polymer and deionized water (DI water), applied voltage, fluid velocity, and concentration of the titanium precursor. For a rheological study, viscosity was observed using a viscometer. The morphology, diameter, and properties of the nanofibers were measured and compared by field emission scanning electron microscope (FE-SEM), thermogravimetric analysis (TGA), and X-ray diffractometer (XRD).

## 2. Materials and Methods

PVP (molecular weight 8000 g/mol, 58,000 g/mol, 1,300,000 g/mol) was purchased from Alfa Aesar Korea Co., Ltd. (Incheon, Korea). Ethanol (EtOH) was purchased from Samchun Chemical Co., Ltd. (Seoul, Korea). Acetyl acetone (ACAC) and titanium 4-isopropoxide (TTIP) were purchased from Junsei Co., Ltd. (Tokyo, Japan).

To compare the microstructure of different molecular weights of PVP, electrospinning solutions containing 10 wt% PVP with three different molecular weights (8000 g/mol, 58,000 g/mol, and 1,300,000 g/mol) in EtOH were prepared. After solution preparation, the samples were loaded into a plastic syringe and connected to a needle. The spinning needle diameter was about 0.337 mm, the fluid velocity was maintained at 0.2 mL/h, and a voltage of 20 kV was applied to the spinning solution by a direct current power supply. To compare the microstructure with different PVP concentrations, 5 ethanol solutions containing 2~10 wt% PVP (1,300,000 g/mol) samples were loaded into a plastic syringe and connected to the needle. The spinning needle diameter was about 0.337 mm, the fluid velocity was maintained at 0.2 mL/h, and a voltage of 20 kV was applied to the spinning solution. Viscosities of PVP solutions were measured and compared by viscometer (LVT, AMETEK Brookfield, Middleboro, MA, USA) with different molecular weight and concentrations.

To compare the microstructure for different DI water concentrations, a 10:0~0:10 ratio of EtOH and DI water solution containing 10 wt% PVP (1,300,000 g/mol) was electrospun under the following conditions: 20 kV applied voltage, 0.337 mm needle diameter, and 0.2 mL/h fluid velocity. Viscosities were measured and compared with a viscometer. To compare the microstructure with different applied voltages and fluid velocities, PVP nanofibers were prepared under the conditions of 10 wt% PVP (1,300,000 g/mol) in ethanol and 0.337 mm nozzle inner diameter by varying the process voltage from 10~20 kV and the flow rate from 0.2~1.0 mL/h.

Raw PVP (1,300,000 g/mol), PVP nanofibers, and PVP/TiO_2_ nanofibers were compared by TGA (STA 409, Q500, NETZSCH Korea Co., Ltd., Paju, Korea) analysis in a nitrogen atmosphere up to 600 °C, and PVP/TiO_2_ nanofibers up to 600 °C in nitrogen and oxygen atmospheres. PVP nanofibers were prepared by electrospinning under conditions of 10 wt% PVP (1,300,000 g/mol) in ethanol, a 0.337 mm nozzle inner diameter, 20 kV voltage, and 1.0 mL/h fluid velocity; and PVP/TiO_2_ nanofibers were prepared by electrospinning under conditions of 5 wt% TTIP, 5 wt% ACAC and 10 wt% PVP in 8:2 mixtures of EtOH and DI water, a 0.337 mm nozzle inner diameter, 20 kV voltage, and 0.2 mL/h fluid velocity. The heat treatment temperature was determined through TGA analysis and heat treatment was performed at 450 °C for 3 h in an air atmosphere. After heat treatment and crystallization, the crystal structures were analyzed by XRD (AXS-D8, Bruker Korea Co., Ltd., Gyeonggi, Korea).

To observe the changes in the microstructure of PVP/TiO_2_ nanofibers by the difference in fluid velocity and titanium precursor concentration, PVP/TiO_2_ nanofibers were fabricated by electrospinning under conditions of 5 wt% TTIP, 5 wt% ACAC and 10 wt% PVP (1,300,000 g/mol) in 8:2 mixtures of EtOH and DI water, a 0.337 mm nozzle inner diameter, 20 kV voltage, and 0.1~1.0 mL/h fluid velocity. By varying the weight ratio of TTIP and ACAC to 5:5, 3:7, and 1:9, an 8:2 ratio of ethanol and DI water solution containing 10 wt% TTIP/ACAC and 10 wt% PVP (1,300,000 g/mol) was used at a voltage of 20 kV, fluid velocity of 0.1 mL/h, and nozzle inner diameter of 0.337 mm under conditions of electrospinning.

After electrospinning and heat treatment, microstructural changes were measured and compared before and after heat treatment by FE-SEM (Inspect F, FEI Korea Co., Ltd., Gyeonggi, Korea).

## 3. Results and Discussion

**Figure 1:**
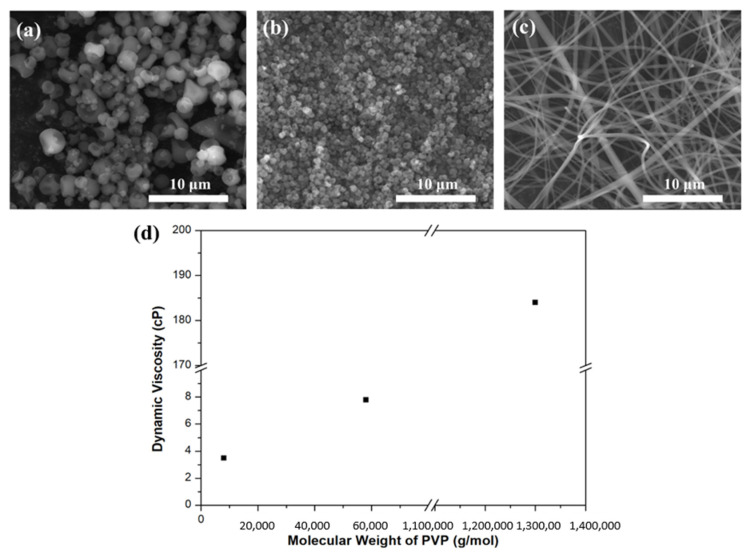
FE-SEM images of nanoparticles and nanofibers with different PVP molecular weights: (**a**) 8000 g/mol, (**b**) 58,000 g/mol, (**c**) 1,300,000 g/mol, and (**d**) dynamic viscosity.

Figure 1 shows FE-SEM images of nanoparticles, nanofibers, and viscosities with different PVP molecular weights of electrospinning solution. When the electrospinning solution contained PVP with a molecular weight of 8000 g/mol and 58,000 g/mol, it formed a shape close to spherical particles, and their average diameters were about 1850.8 nm and 527.6 nm, respectively. When the electrospinning solution contained PVP with a molecular weight of 1,300,000 g/mol, it formed in the shape of nanofibers, and their average diameter was about 339.0 nm. The viscosities of PVP solution of 8000 g/mol, 58,000 g/mol, and 1,300,000 g/mol PVP molecular weight were 3.5 cP, 7.8 cP, and 184.0 cP, respectively.

**Figure 2:**
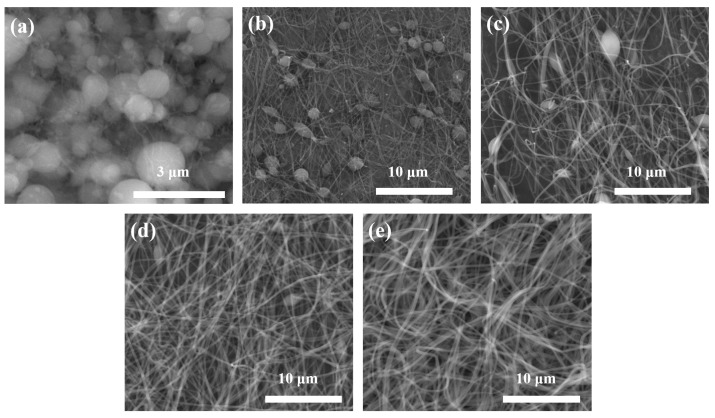
FE-SEM images of mixed microstructures with beads and nanofibers, with different PVP concentrations: (**a**) 2 wt%, (**b**) 4 wt%, (**c**) 6 wt%, (**d**) 8 wt%, and (**e**) 10 wt%.

**Figure 3:**
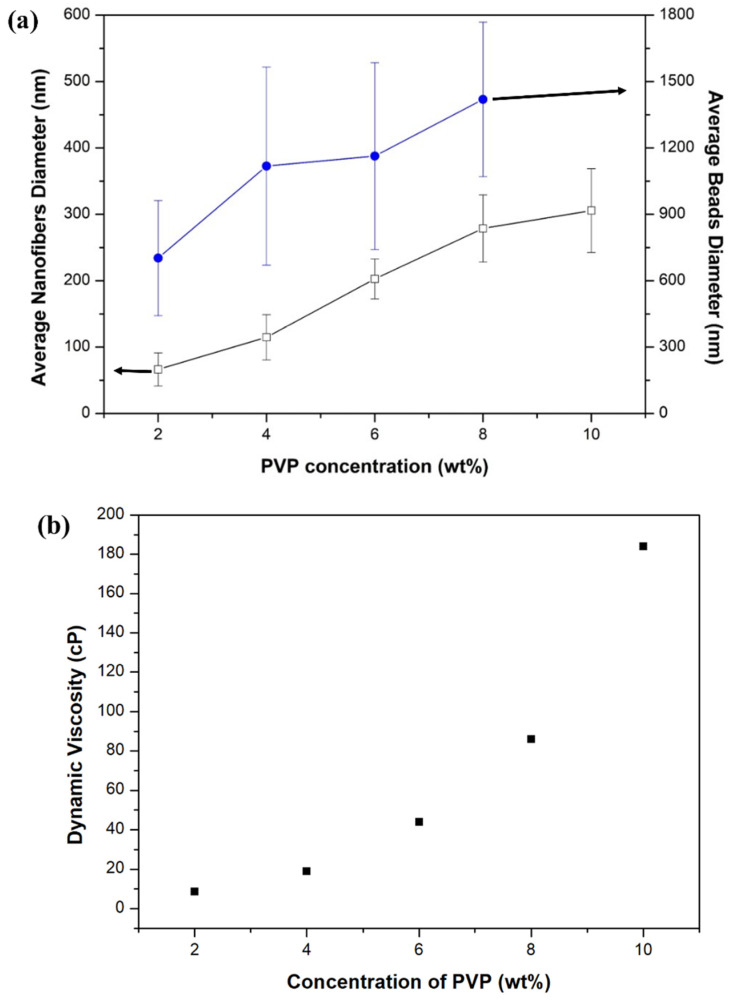
(**a**) Average diameter of beads and nanofibers, (**b**) viscosities with different PVP concentrations.

Figure 2 shows FE-SEM images of mixed microstructures with beads and nanofibers as the PVP (1,300,000 g/mol) concentration changes from 2 wt% to 10 wt%, and Figure 3 shows the average diameters of the beads and nanofibers, and viscosities. In a PVP concentration range of 2 wt% to 8 wt%, beads formed, and their number decreased with increasing PVP concentration. The average diameters of the beads in 2 wt%, 4 wt%, 6 wt%, and 8 wt% PVP concentrations were 702.3 nm, 1118.0 nm, 1163.1 nm, and 1419.3 nm, respectively. The average diameters of the nanofibers increased with increased PVP concentration. The values at 2 wt%, 4 wt%, 6 wt%, 8 wt%, and 10 wt% PVP concentration were 66.5 nm, 114.8 nm, 202.8 nm, 278.7 nm, and 305.7 nm, respectively. The viscosities of PVP solution at 2 wt%, 4 wt%, 6 wt%, 8 wt%, and 10 wt% PVP concentration were 8.5 cP, 19.0 cP, 44.0 cP, 86.0 cP, and 184.0 cP, respectively. The viscoelasticity of the polymer solution competes with the Rayleigh instability and is conventionally determined by the polymer concentration or molecular weight. Increasing the polymer concentration or entanglements in the solution causes particle electrospray to transit to fiber electrospinning, with beads-on-strings morphologies, as a result of the incomplete suppression of the Rayleigh instabilities [40]. The molecular weight of the polymer has a significant effect on the rheological and electrical properties, such as viscosity, surface tension, conductivity, and dielectric strength. This is another important solution parameter that affects the morphology of electrospun fibers, and generally high molecular weight polymer solutions have been used in electrospinning as they provide the desired viscosity for fiber generation. It has been observed that a solution with too low a molecular weight tends to form beads rather than fibers, and a high molecular weight solution gives fibers with larger average diameters. The molecular weight of the polymer reflects the number of entanglements of the polymer chains in a solution, and thus solution viscosity. Chain entanglement plays an important role in the process of electrospinning. To fabricate nanofiber, the entanglement of the polymer chains must be of sufficient viscosity to create a uniform jet, otherwise particles and bead shapes will be produced [26,41,42].

**Figure 4:**
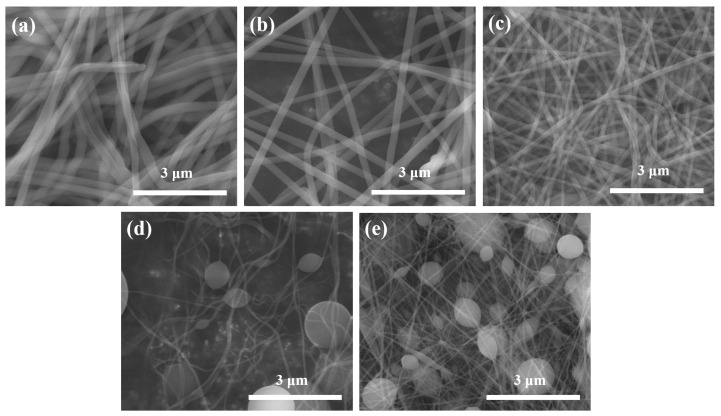
FE-SEM images of mixed microstructures with beads and nanofibers in various weight ratios of EtOH to DI water in solvents (**a**) 10:0, (**b**) 8:2, (**c**) 5:5, (**d**) 2:8, and (**e**) 0:10.

**Figure 5:**
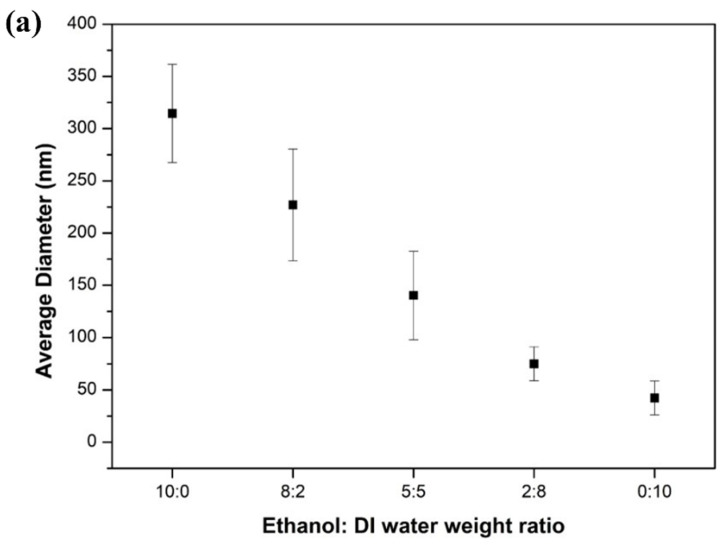

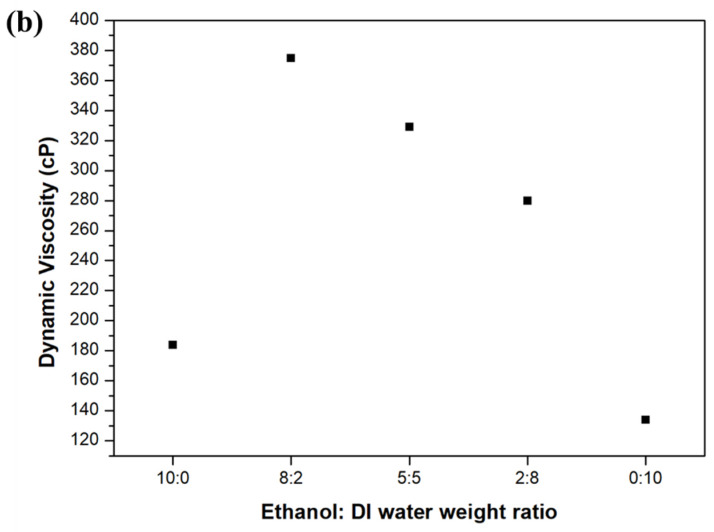
(**a**) Average diameter of nanofibers, and (**b**) viscosities with different EtOH and DI water weight ratios in solvents.

Figure 4 shows FE-SEM images of mixed microstructures with beads and nanofibers with various weight ratios of EtOH to DI water in solvents, and Figure 5 shows their average diameters and viscosities of PVP solution. The diameter of the nanofibers decreased with an increasing ratio of DI water in the solvents from 0 to 10. The average diameters of nanofibers in 10:0, 8:2, 5:5, 2:8, and 0:10 were 314.6 nm, 227.0 nm, 140.3 nm, 75.0 nm, and 42.3 nm, respectively. The viscosities of PVP solution in 10:0, 8:2, 5:5, 2:8, and 0:10 of solution weight ratio were 184.0 cP, 375.0 cP, 329.0 cP, 280.0 cP, and 134.0 cP, respectively. When the ratio of DI water was increased, the diameters of the nanofibers were decreased. When the ratio of DI water was greater than 5:5, beads formed, and the number of beads increased with an increasing amount of DI water. Water has a higher dielectric constant and dipole moment than ethanol, which means the jet using water as a solvent is subjected to a stronger elongation force. On the other hand, the dryness of the electrospun fibers increases with the decreasing density and boiling point of the solvents. The evaporation of the solvent systems richer in water was much slower than those richer in ethanol, due to the higher density and boiling point of water. Thus, the viscoelastic force acting on the charged jet rapidly increased with increasing ethanol content in a mixed solvent, due to the extremely rapid solvent evaporation. As soon as the viscoelastic force exceeded the Coulomb force, the jets could not be stretched any further, and the fibers were subjected to a smaller elongation force for a shorter time [43,44].

**Figure 6:**
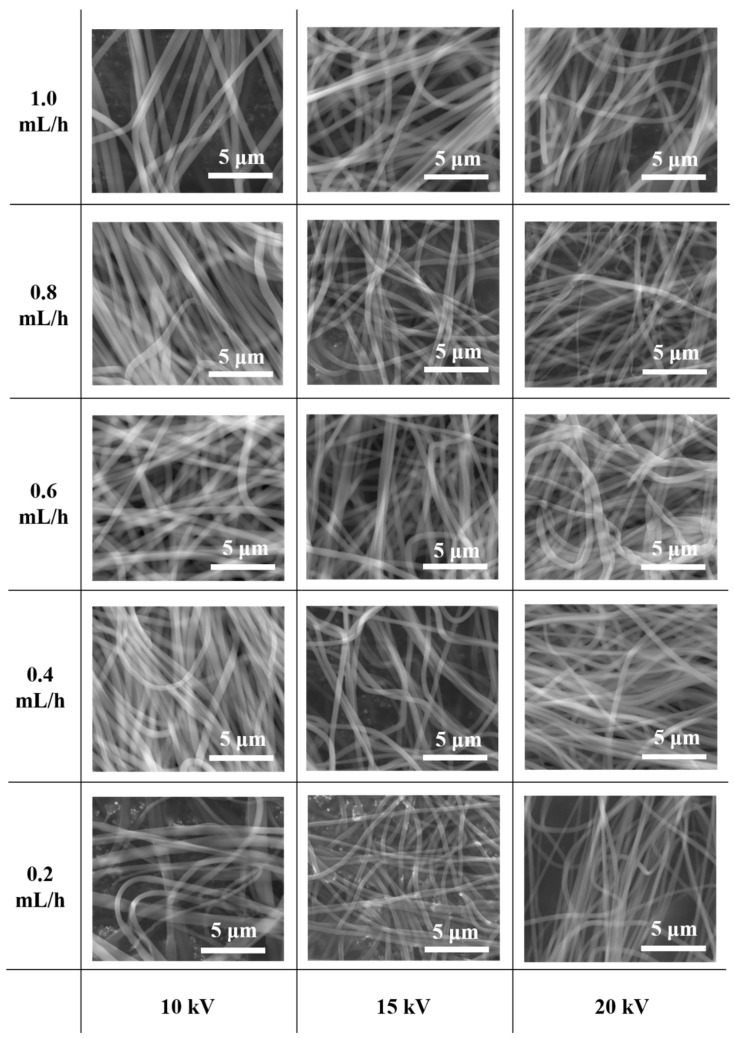
FE-SEM images of electrospun nanofibers with different voltages and fluid velocities.

**Figure 7:**
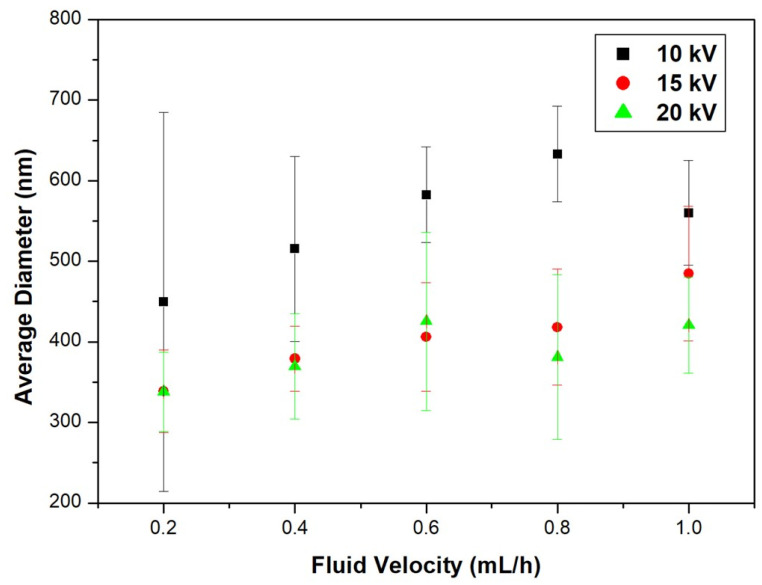
Average diameter of nanofibers with different voltages and fluid velocities.

Figure 6 shows FE-SEM images of electrospun nanofibers with different voltages and fluid velocities, and Figure 7 shows their average diameters. At an applied voltage of 10 kV, the average diameters of the nanofibers in 0.2 mL/h, 0.4 mL/h, 0.6 mL/h, 0.8 mL/h, and 1.0 mL/h were 449.7 nm, 515.4 nm, 582.4 nm, 633.0 nm, and 560.0 nm, respectively. At an applied voltage of 15 kV, the average diameters of the nanofibers in 0.2 mL/h, 0.4 mL/h, 0.6 mL/h, 0.8 mL/h, and 1.0 mL/h were 338.6 nm, 379.2 nm, 406.0 nm, 418.2 nm, and 484.8 nm, respectively. At an applied voltage of 20 kV, the average diameters of the nanofibers in 0.2 mL/h, 0.4 mL/h, 0.6 mL/h, 0.8 mL/h, and 1.0 mL/h were 338.1 nm, 369.5 nm, 425.7 nm, 381.0 nm, and 420.7 nm, respectively. When the voltages were 15 kV and 20 kV, the diameter change of the nanofibers was not large, but the diameter increased significantly as the voltage decreased to 10 kV. In the electrospinning process, above a critical voltage, the effect of voltage on the formation of fibers was small. However, at lower voltage, the instability resulting from the electric field was small and the charge stress contributing to the elongation of the fibers occurring simultaneously with solvent volatilization was small. As the fluid velocity decreased, the average diameter of the nanofibers tended to decrease.

**Figure 8:**
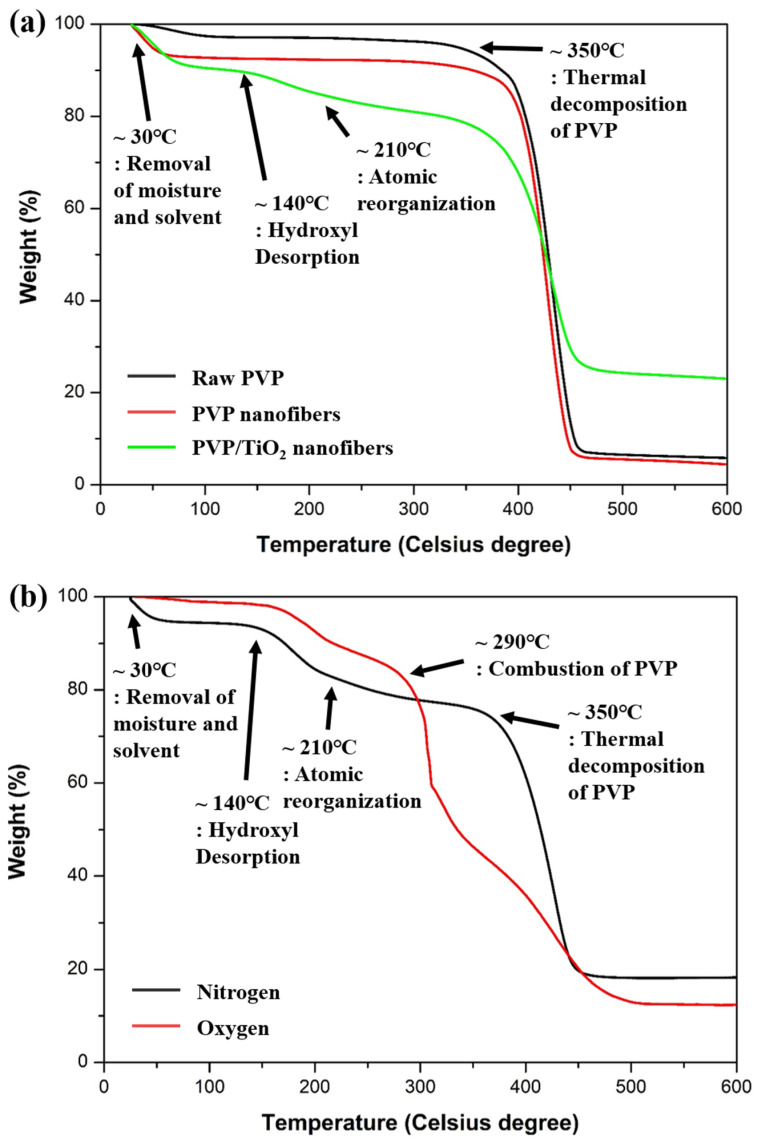
TGA curves of (**a**) raw PVP, PVP nanofibers, PVP/TiO_2_ nanofibers in a nitrogen atmosphere, and (**b**) PVP/TiO_2_ nanofibers in a nitrogen and oxygen atmosphere.

**Figure 9:**
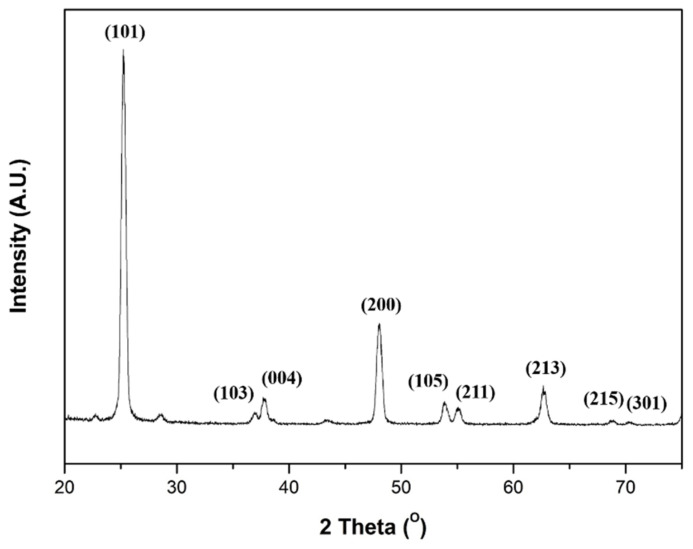
XRD spectrum of heat-treated PVP/TiO_2_ nanofibers.

Figure 8 shows TGA curves of raw PVP, PVP nanofibers, PVP/TiO_2_ nanofibers in a nitrogen atmosphere, and PVP/TiO_2_ nanofibers in a nitrogen and oxygen atmosphere. Compared with raw PVP, electrospun nanofibers contained moisture and organic solvents, so the initial weight reduction was large. PVP/TiO_2_ nanofibers containing TTIP and ACAC showed weight loss due to desorption of hydroxyl groups from about 140 °C, and crystallization appeared from 210 °C. In all the samples, the largest weight loss was observed in the range of about 350 °C to 450 °C, due to PVP decomposition. In addition, in a nitrogen atmosphere, PVP was thermally decomposed, and about 5 wt% remained, while PVP/TiO_2_ nanofibers containing TTIP and ACAC showed a weight reduction of about 75 wt%. In both the nitrogen and oxygen atmospheres, the weight initially decreased due to volatilization of the water and organic solvents, and the weight decreased from about 140 °C due to desorption of hydroxyl groups, while crystallization occurred from 210 °C. In the nitrogen atmosphere, the weight was reduced by the thermal decomposition of PVP, and in the oxygen atmosphere, the PVP was combusted at the same time as the thermal decomposition, and the weight decreased. These transformations agreed with the literature [45,46,47]. Figure 9 shows the XRD spectrum of heat-treated PVP/TiO_2_ nanofibers. The peaks of TiO_2_ anatase were identified.

**Figure 10:**
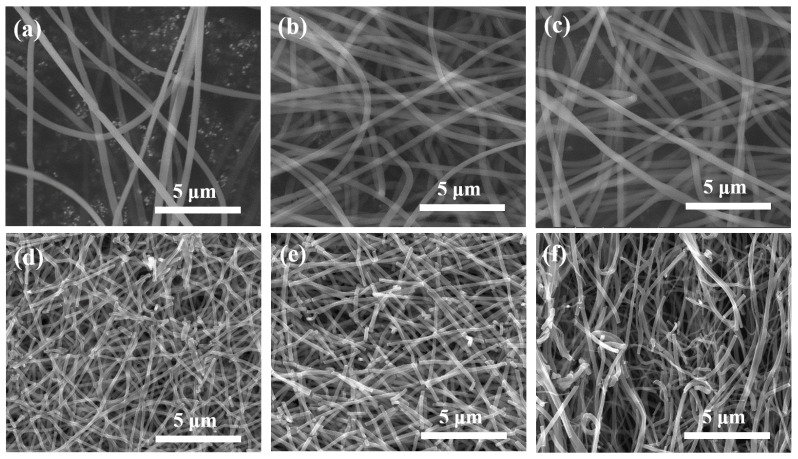
FE-SEM images of PVP/TiO_2_ nanofibers with different fluid velocities before (**a**) 0.1 mL/h, (**b**) 0.5 mL/h, (**c**) 1.0 mL/h and after heat treatment (**d**) 0.1 mL/h, (**e**) 0.5 mL/h, (**f**) 1.0 mL/h.

**Figure 11:**
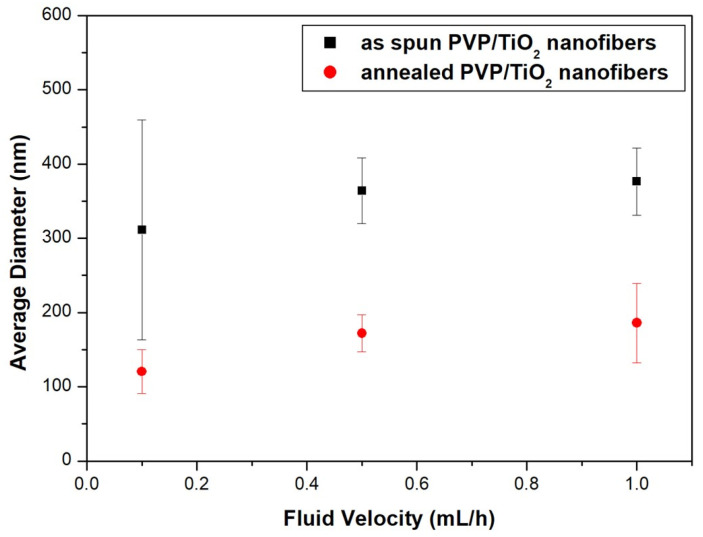
Average diameter of PVP/TiO_2_ nanofibers with different fluid velocities before and after heat treatment.

Figure 10 shows FE-SEM images of PVP/TiO_2_ nanofibers with different fluid velocities before and after heat treatment, and Figure 11 shows their average diameter. The average diameter of nanofibers decreased with the fluid velocity. Before heat treatment, the average diameter of the nanofibers at fluid velocities of 0.1 mL/h, 0.5 mL/h, and 1.0 mL/h were 311.4 nm, 364.1, and 376.5 nm, respectively. The values after heat treatment were decreased to 120.5 nm, 172.1, and 186.0 nm due to calcination of the PVP.

**Figure 12:**
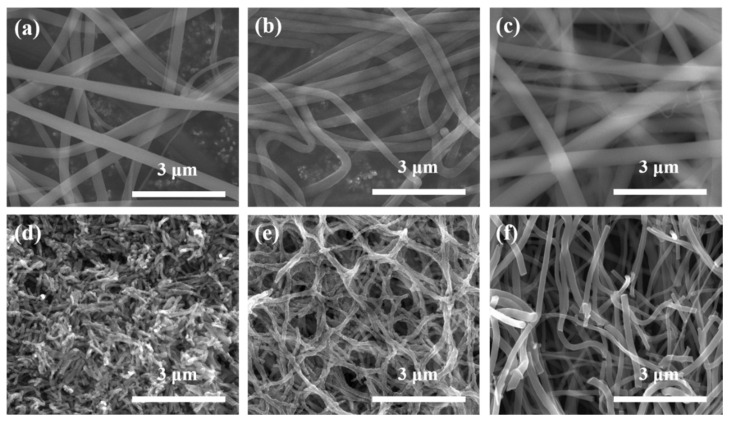
FE-SEM images of PVP/TiO_2_ nanofibers with different weight ratios of TTIP and ACAC before (**a**) 1:9, (**b**) 3:7, (**c**) 5:5 and after annealing (**d**) 1:9, (**e**) 3:7, (**f**) 5:5.

**Figure 13:**
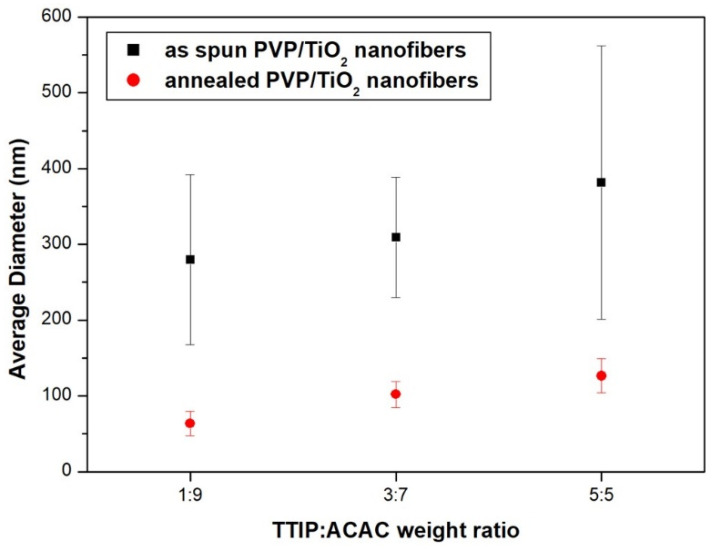
Average diameter of PVP/TiO_2_ nanofibers with different weight ratios of TTIP and ACAC before and after heat treatment.

Figure 12 shows FE-SEM images of PVP/TiO_2_ nanofibers with different weight ratios of TTIP and ACAC before and after annealing, and Figure 13 shows their average diameters. Before heat treatment, the average diameters of the nanofibers at TTIP weight ratios of 1:9, 3:7, and 5:5 were 279.7 nm, 309.3 nm, and 381.5 nm, respectively. The values after heat treatment were 63.5 nm, 102.2 nm, and 126.6 nm. The reduction was due to the calcination of PVP through heat treatment. The average diameters decreased with decreasing TTIP weight ratio.

Many process parameters can affect the microstructure of PVP/TiO_2_ nanofibers. Moreover, the microstructure of TiO_2_ nanofibers can affect their optical, mechanical, and photocatalytic properties [48,49,50,51].

## 4. Conclusions

To fabricate various nanostructured PVP and PVP/TiO_2_ nanofibers by electrospinning, we controlled some process parameters, namely, the molecular weight and concentration of PVP, DI water weight ratio in the solvent, applied voltage, fluid velocity, and TTIP weight ratio. With an increase in the DI water ratio and a decrease in the molecular weight and concentration of PVP, the average diameters of the PVP nanofibers and spherical shape formations decreased. With a decrease in fluid velocity and an increase in the applied voltage, the average diameter of the PVP nanofibers decreased. The average diameter was reduced due to the calcination of PVP through heat treatment. The diameter of the PVP/TiO_2_ nanofibers was decreased by a decrease in the fluid velocity and TTIP ratio. We succeeded in controlling the nanostructure of the PVP/TiO_2_ nanofibers using the process parameters, and the resulting nanofibers are nanostructures appropriate for many applications, such as photocatalysts, solar cells, sensors, and filters.
